# Phase II study of intraperitoneal recombinant interleukin-12 (rhIL-12) in patients with peritoneal carcinomatosis (residual disease < 1 cm) associated with ovarian cancer or primary peritoneal carcinoma

**DOI:** 10.1186/1479-5876-5-66

**Published:** 2007-12-12

**Authors:** Renato Lenzi, Robert Edwards, Carl June, Michael V Seiden, Michael E Garcia, Michael Rosenblum, Ralph S Freedman

**Affiliations:** 1 Department of GI Medical Oncology, The University of Texas M.D. Anderson Cancer Center, Houston, Texas, USA; 2 Department of Obstetrics & Gynecology, University of Pittsburgh Medical Center, Pittsburgh, PA, USA; 3 Abramson Cancer Center, University of Pennsylvania, Philadelphia, PA, USA; 4 Fox Chase Cancer Center, Philadelphia, PA, USA; 5 Department of Gynecologic Oncology, The University of Texas M.D. Anderson Cancer Center, Houston, Texas, USA; 6 Department of Experimental Therapeutics, The University of Texas M.D. Anderson Cancer Center, Houston, Texas, USA

## Abstract

**Background:**

Pharmacokinetic advantages of intraperitoneal (IP) rhIL-12, tumor response to IP delivery of other cytokines as well as its potential anti-angiogenic effect provided the rationale for further evaluation of IPrhIL-12 in patients with persistent ovarian or peritoneal carcinoma.

**Methods:**

A phase 2 multi-institutional trial (NCI Study #2251) of IP rIL-12 (300 nanogram/Kg weekly) was conducted in patients with ovarian carcinoma or primary peritoneal carcinoma.

Patients treated with primary therapy for ovarian cancer who had no extraabdominal/parenchymal disease or bulky peritoneal disease were eligible. Four to 8 weeks from last chemotherapy, eligible patients underwent a laparotomy/laparoscopy. Patients with residual disease ≤ 1 cm were registered for the treatment phase 2–5 weeks post surgery. The effect of IP rIL-12 on the expression of TNFα , INFα , IL-10, IP-10, IL-8, FGF, VEGF was also studied.

**Results:**

Thirty-four patients were registered for the first screening phase of the study. Median age was 56.6 years (range: 31–71); 12 completed the second phase and were evaluable for response/toxicity. Performance scores of IL-12 treated patients were 0 (11 pts) and 1 (1 pt). There were no treatment related deaths, peritonitis or significant catheter related complications. Toxicities included grade 4 neutropenia (1), grade 3 fatigue (4), headache (2), myalgia (2), non-neutropenic fever (1), drug fever (1), back pain (1), and dizziness (1). The best response observed was SD. Two patients had SD and 9 had PD, and 1 was evaluable for toxicity only.

Peritoneal fluid cytokine measurements demonstrated a ≥ 3 fold relative increase post-rhIL-12: IFN-γ, 5/5 pts; TNF-α , 1/5; IL-10, 4/5; IL-8, 5/5; and VEGF, 3/5. IP10 levels were increased in 5/5 patients. Cytokine response profiles suggest either NK or T-cell mediated effects of IP rhIL-12. Cytokine/chemokine results also suggest a pleiotropic response since proteins with potential for either anti-tumor (IFN-γ , IP-10) or pro-tumor growth effects (VEGF, IL-8) were detected.

**Conclusion:**

IP IL-12 can safely be administered at this dose and schedule to patients after first line chemotherapy for ovarian/peritoneal carcinoma. The maximum response was stable disease. Future IP therapies with rhIL-12 will require better understanding and control of pleiotropic effects of IL-12.

## Introduction

The limited success of systemic chemotherapy for the treatment of Mullerian type carcinomas involving the peritoneum has promoted interest in the intraperitoneal (IP) route of administration of therapeutic agents for this condition. Advantages of IP administration of biologics include a lower clearance rate relative to plasma, and higher concentration in IP fluids relative to plasma, producing a possible direct effect on the tumor [1]. Pathological responses have been observed following intraperitoneally administered rIFN-α [2,3], rIFN-γ [4], and rIL-2 [5,6]. IL-12 a large heterodimer, is a powerful inducer of the production of IFN-γ and TNF-α, cytotoxic natural killer and T-lymphocytes and of lymphokine-activated killer cells. Additionally, IL-12 has anti-angiogenic activity in animal studies [7,8].

In a Phase I study [9] of IP IL-12 in patients with peritoneal carcinomatosis from Mullerian and gastrointestinal primary tumors who were not responding to standard chemotherapy treatment, rhIL-12 was given weekly on 4 week cycles. Patients were evaluated for response every two cycles. Patients with stable disease or responding were treated for up to 6 months. Dose levels ranged from 3 to 600 ng/kg. Of the 26 patients entered in the phase1 study, none were removed because of dose-limiting toxicities. The only grade 4 toxicities were 2 instances of grade 4 lymphopenia. DLT was reached at 600 ng/kg. DLT was indicated by elevation of transaminases that did not return to baseline by the scheduled start of next treatment cycle. In that study one patient experienced a CR laparoscopically documented and 7 patients had stable disease. Based on these results the dose of 300 ng/kg administered weekly was selected for the phase 2 study. The previous study also included detailed pharmacokinetics and pharmacodynamics [9].

## Methods

This phase 2 multi-institutional clinical trial of intraperitoneal recombinant interleukin-12 (Genetics Institute, Cambridge, MA, U.S.A.)  in patients with epithelial ovarian carcinoma or primary peritoneal carcinoma with residual disease less than 1 cm in maximum diameter after cancer reductive surgery and platinum based chemotherapy had the primary goals to assess response rate by a laparoscopy or laparotomy, to determine the qualitative and quantitative toxicity of interleukin 12, and to measure progression-free survival.

Secondary objectives included the measurement of changes in posttreatment cytokine profiles, the effect of intraperitoneal interleukin-12 on the expression of VEGF, FGF_2_, and IL-8 as surrogate markers of angiogenesis. The study was approved by the IRBs at University of Texas M.D. Anderson Cancer Center, Massachusetts General Hospital, University of Louisville and University of Pennsylvania.

Eligibility criteria included a pathologic diagnosis of peritoneal carcinomatosis secondary to ovarian or primary peritoneal tumors, surgically documented peritoneal disease of < 1 cm after primary treatment including surgery and platinum based chemotherapy, adequate bone marrow, liver, renal function, successful placement of IP catheter, Zubrod performance status of ≤ 1.

Exclusion criteria included intra-abdominal disease of ≥ 1 cm in max diameter, any extra-abdominal or parenchymal disease, and conditions with the potential to negatively affect immunocompetence (such as chronic use of steroids, HIV, hepatitis B or C).

The study plan included the initial enrollment of patients who had received primary therapy for their cancer and who had no evidence of extraabdominal/parenchymal disease or of peritoneal disease ≥ 1 cm in max diameter by imaging studies (CT or MRI). After 4–8 weeks from last chemotherapy the patients underwent a laparotomy/laparoscopy. Patients with residual disease meeting the above criteria were then registered a second time for the treatment phase of the study 2–5 weeks after the laparotomy/laparoscopy. The study was originally powered to show a greater than 20% response rate with 30 patients.

Peritoneal fluid samples were stored at -20°C and measured for IL-12p70, INF-γ, TNF-α, IL-8, VEGF and IL-10 using the Luminex Multiplex assay and ELISA at M. D. Anderson's Human Cancer Immunology Research Facility using methods previously described [10]. Briefly, the multiplex bead immunoassay is a multiplex assay designed to work with the Luminex 100 analyzer (Luminex, Austin, TX), associated software, and fluorescently encoded microspheres. The minimum detectable levels of each cytokine with this method were (pg/ml): TNF-α, 8.5 ng/ml; IL12p70, 3.5 ng/ml; INFγ, 0.8 ng/ml; VEGF, 24.6 ng/ml; IL10, 1.9 ng/ml; IL8, 1.6 ng/ml; and IP10, 3.2 ng/ml.

## Results

Thirty-four patients were registered for the first phase of the study. Of those, one subsequently declined the procedure. Fourteen had a negative second look laparoscopy. Nineteen patients had a positive second look procedure. Of those, 3 had residual disease of > 1 cm and were not eligible for the second phase of the study. In two, a peritoneal catheter could not be placed and 14 were registered for the second phase. Of those 14 patients, 2 were subsequently determined to be ineligible because of an inadequate blood count or because of a treatment-free interval that exceeded the permitted range. Demographics included: 31 white, 2 black, and 1 Asian; the median age was 56.6 years (range: 31–71).

Of the 12 evaluable patients, 11 were evaluable for response and toxicity; one was evaluable for toxicity only because she was treated for only one month. PS was Zubrod 0 (11 pts) and 1 (1 pt).

Pathological tumor types of evaluable patients included: serous carcinoma (11 pts) and adenocarcinoma (1 pt).

### Response and survival

The best response observed was SD. Two patients had SD and 9 had PD. One patient who was not adequately treated for response evaluation developed neutropenia after 1 week of treatment that did not resolve in time for further dosing. Due to the above response rate (0/12), the protocol was closed to accrual. Of the 14 treated patients, 10 are currently alive and 4 have expired. Five of the patients had a third look laparotomy, the remainder had PD by radiological criteria (CT) and were therefore not eligible for third look. At 16+/-2 weeks, 1 of 5 pts had no change, 4 had PD. One patient with no change by CT declined third look laparotomy, 1 pt had PD in one isolated diaphragmatic focus that was subsequently treated with Doxil for 18 cycles and eventually surgically completely resected. A CT at 3 years demonstrated progression at multiple sites.

#### Number of courses

Thirty-eight courses of therapy were administered to 12 patients (4 weeks = 1 course, 1 IL-12 IP injection/week). These represented a mean and range of 10.75 (1–16) weeks of treatment.

#### Toxicity

There were no treatment related deaths. There were no episodes of peritonitis or other catheter-related complications. Grade 4 toxicities were represented by 1 instance of grade 4 neutropenia requiring removal from study, and grade 3 toxicities of fatigue (4), headache (2), myalgia (2), non-neutropenic fever (1), drug fever (1), back pain (1), dizziness (1).

#### Peritoneal fluid cytokine levels

Serial multiplex ELISA assays for IL-12p70, IFN-γ, TNF-α, IL-10, IL-8, VEGF, and IP-10, were performed on peritoneal fluid samples obtained from 5 patients following the first injection of IP rhIL-12 (Table 1). Pretreatment IL-12p70 values were close to the limit of detection in 5 of 5 patients (8.5, range 6.1–8.7). Peak levels of IL-12p70 were detected at 6 hours and then slowly declined to 48 hours. Greater than 3-fold elevation in the levels of the following cytokines/chemokines were detected post-treatment: IFN-γ, 5/5; TNF-α, 1/5; IL-10, 4/5; VEGF, 3/5; IL-8, 5/5; and IP-10, 5/5.

**Table 1:** Cytokine levels (pg/ml) pre and post-rhIL-12 infusion

**Accession Number**	**Sampling Timepoint**	***Sample ID***	***Results (pg/ml)***						
			***TNF-α***	***IL-12 p70***	***INF-γ***	***VEGF***	***IL-10***	***IL-8***	***IP-10***
5	Baseline	**1**	15.3	6.7	< 0.78	< 2.30	5.9	38.6	7.6
	6 Hr	**2**	16.1	4973.2	1.9	56.4	18.8	270.0	191.6
	24 Hr	**3**	15.7	4722.9	38.1	33.8	35.2	72.7	263.8
	48 Hr	**4**	18.7	8004.8	215.9	74.1	80.5	213.1	303.8
	Baseline	**5**	105.4	7.6	1.0	36.4	36.5	190.0	12.9
	6 Hr	**6**	34.6	11040.0	140.1	348.8	3686.9	1254.7	460.2
	24 Hr	**28**	70.1	3789.5	232.5	238.8	553.0	3897.2	367.2
	48 Hr	**7**	43.4	1085.4	198.9	178.8	187.1	6363.6	354.0

11	Baseline	**8**	16.1	7.1	< 0.78	48.4	18.4	67.7	7.9
	6 Hr	**9**	17.4	5380.8	< 0.78	69.7	39.9	122.8	251.2
	24 Hr	**10**	74.1	5217.5	56.8	87.3	72.2	876.7	354.3
	48 Hr	**11**	48.0	934.2	220.0	82.6	30.1	128.0	1153.9

10	Baseline	**12**	16.6	6.1	1.0	89.5	5.5	231.7	7.9
	6 Hr	**13**	55.2	25700.0	37.7	447.5	296.5	1577.8	351.3
	24 Hr	**14**	185.3	6580.7	236.3	428.0	90.7	1538.3	429.6
	48 Hr	**15**	56.0	561.2	155.3	379.3	27.3	2923.8	355.6

4	Baseline	**20**	15.3	6.4	< 0.78	< 2.30	3.6	7.2	< 0.95
	6 Hr	**21**	16.1	5937.5	2.2	82.8	4.8	251.3	238.2
	24 Hr	**22**	17.4	5547.5	74.8	69.4	9.7	93.2	258.2
	48 Hr	**23**	35.1	4283.1	504.3	55.7	27.3	124.7	275.7

3	Baseline	**24**	17.4	8.7	10.0	110.3	10.4	492.4	9.0
	6 Hr	**25**	17.4	10214.7	19.5	213.7	15.8	1711.3	229.7
	24 Hr	**26**	40.6	4103.6	184.2	187.9	19.9	3348.5	223.9
	48 Hr	**27**	16.6	30.7	108.8	37.7	3.6	475.5	202.0

## Discussion

Protocol feasibility was less than anticipated, as only 12 of 34 patients who underwent second look laparotomy/laparoscopy were eligible for treatment having residual disease ≤ 1 cm in maximum linear diameter. A larger number of patients had to be screened surgically to determine eligibility for the second registration since many were negative for tumor, had tumors too large for the treatment, or adhesions precluded adequate treatment. These factors cannot be determined accurately utilizing non-surgical approaches. It is also possible that accrual to the study was adversely affected by competing first line studies for ovarian cancer that disallow surgical re-evaluation and because it has become accepted practice that second look does not afford a survival benefit. Our data emphasize the pressing clinical need for novel non-invasive radiologic modalities to identify small residual disease which is optimal for IP therapy. CA125 has performed well as a surrogate for radiological studies [11], but has not been adequately evaluated and validated as a marker for disease progression or response while patients are receiving IP therapy. The catheter and/or endogenous cytokines produced in response to treatment might produce inflammatory effects of treatment that affect systemic CA125 levels.

The study was originally powered to show a greater than 20% response rate with 30 patients. Accrual to the therapy phase was stopped early. However, the results from this study failed to show significant benefit from IP rIL12 at this dose and schedule in any of 11 patients evaluable for response. Ten of 12 treated patients reached the 8 weeks' treatment timeline for reevaluation with no objective responses observed. Only one surgically validated SD was observed out of 5 patients who underwent third look laparotomy/laparoscopy. Cytokine responses indicated either NK or T-cell mediated effects of IP rhIL-12 with results suggesting pleiotropism with endogenous production of immunostimulatory cytokines or chemokines (IFN-γ, IP-10) or tumor-promoting effects (VEGF and IL-8). Elevated levels of IL-10 could be the result of stimulation of either macrophages [12] or certain T-cell subsets such as T-regs although TNF-α levels were only increased in 1 of 5 patients. Insufficient cellular material was available to examine cellular responses. We have, however, in a separate study, shown that tryptophan levels may decrease after IP IL12 and that nitrate levels may increase [13]. This raises a concern since the endogenous production of nitric oxide can be damaging to either tumor cells or immune effector cells.  There is also the possibility that local production of IFN-γ might shift the arginine pathway toward polyamine production which can have a stimulatory effect on tumor metabolism [14].

## Conclusion

rhIL-12 can be safely administered IP to patients with ovarian or primary peritoneal disease and abdominal carcinomatosis.

The true rate of response is difficult to estimate based on the data obtained from this study since the projected accrual was not reached. Since there were 0/12 responses in evaluable patients, the RR would be unlikely to exceed 20% or be strongly supportive of future studies with IP rhIL12. Furthermore, pleiotropic effects of rhIL-12 on different target cell populations could produce mixed pro- and anti-tumor growth effects, suggesting that the use of rhIL-12 may have a limited role in the treatment of ovarian cancer in this setting.

## References

[B1] Dedrick RL, Meyers CE, Bungay PM (1978). Pharmacokinetic rationale for peritoneal drug administration in the treatment of ovarian cancer. Cancer Treatment Reports.

[B2] Berek JS, Hacker NF, Lichtenstein A, Jung T, Spina C, Knox RM, Brady J, Greene T, Ettinger LM, Lagasse LD, Bonnem EM, Spiegel RJ, Zighelboim J (1985). Intraperitoneal recombinant a-interferon for "salvage" immunotherapy in stage III epithelial ovarian cancer:  A gynecologic oncology group study. Cancer Res.

[B3] Willemse PHB, De Vries EGE, Mulder NH, Aalders JG, Bouma J, Sleijfer DT (1990). Intraperitoneal human recombinant interferon alpha-2b in minimum residual ovarian cancer. Eur J Cancer.

[B4] Pujade-Lauraine E, Guastella JP, Colombo N, Devillier P, Francois E, Fumoleau P, Monnier A, Nooy M, Mignot L, Bugat R, Marques C, Mousseau M, Netter G, Maloisel F, Larbaoui S, Brandely M (1996). Intraperitoneal recombinant interferon-gamma in ovarian cancer patients with residual disease at second-look laparotomy.. J Clin Oncol.

[B5] Edwards RP, Gooding W, Lembersky BC, Colonello K, Hammond R, Paradise C, Koal CD, Kunscher AJ, Baldisseri M, Kirkwood JM, Herberman RB (1997). Comparison of toxicity and survival following intraperitoneal recombinant interleukin-2 for persistent ovarian cancer after platinum: twenty-four-hour versus 7-day infusion. J Clin Oncol.

[B6] Steis RG, Urba WJ, VanderMolen LA, Bookman MA, Smith JW, Clark JW, Miller RL, Crum ED, Beckner SK, McKnight JE (1990). Intraperitoneal lymphokine-activated killer cell and interleukin-2 therapy for malignancies limited to the peritoneal cavity. J Clin Oncol.

[B7] Sgadari C, Angiolillo AL, Tosato G (1996). Inhibition of angiogenesis by interleukin-12 is mediated by the interferon-inducible protein 10. Blood.

[B8] Voest EE, Kenyon BM, O'Reilly MS (1995). Inhibition of angiogenesis in vivo by interleukin-12. J Natl Cancer Inst.

[B9] Lenzi R, Rosenblum M, Verschraegen C, Kudelka AP, Kavanagh JJ, Hicks ME, Lang EA, Nash MA, Levy LB, Garcia ME, Platsoucas CD, Abbruzzese JL, Freedman RS (2002). Phase I study of intraperitoneal rhIL-12 in patients with mullerian carcinoma, gastrointestinal primary malignancies and mesothelioma. Clinical Cancer Research.

[B10] Gordon IO, Freedman RS (2006). Defective antitumor function of monocyte-derived macrophages from epithelial ovarian cancer patients. Clin Cancer Res.

[B11] FDA (2006). Meeting Summary April 26, 2006: Bethesda, MD..

[B12] Loercher AE, Nash MA, Kavanagh JJ, Platsoucas CD, Freedman RS (1999). Identification of an IL-10 producing HLA-DR-negative monocyte subset in the malignant ascites of patients with ovarian carcinoma that inhibits cytokine protein expression and proliferation of autologous T cells. J Immunol.

[B13] Melichar B, Lenzi R, Rosenblum M, Kudelka AP, Kavanagh JJ, Melicharova K, Templin S, Garcia ME, Abbruzzese JL, Freedman RS (2003). Intraperitoneal fluid neopterin, nitrate, and tryptophan after regional administration of interleukin-12. J Immunother.

[B14] Melichar B, Hu W, Patenia R, Melicharova K, Gallardo ST, Freedman RS (2003). rIFN-gamma-mediated growth suppression of platinum-sensitive and -resistant ovarian tumor cells lines not dependent upon arginase inhibition. J Translational Medicine.

